# Prevalence and predictors of risk factors for cardiovascular diseases among women aged 15–49 years across urban and rural India: findings from a nationwide survey

**DOI:** 10.1186/s12905-023-02869-0

**Published:** 2024-01-28

**Authors:** Shyambhavee Behera, Rahul Sharma, Kartikey Yadav, Pragti Chhabra, Milan Das, Sonu Goel

**Affiliations:** 1 Department of Community Medicine, Hamdard Institute of Medical Sciences and Research, New Delhi, India; 2 Department of Community Medicine, University College of Medical Sciences & GTB Hospital, Delhi, India; 3 International Institute for Population Sciences, Mumbai, Maharashtra India; 4 School of Medicine, University of Limerick, Limerick, Ireland

**Keywords:** Cardiovascular diseases, Women, NCD risk factors, National survey, Non-communicable diseases

## Abstract

**Background:**

Women’s health is usually looked upon in terms of their reproductive health. However, cardio-vascular diseases are one of the leading causes of death and disability among women, globally as well as in India. Risk factors of today can be disease of tomorrow. Gradience in level of epidemiological transition is observed across different states. The study aims to estimate the national and regional prevalence, and sociodemographic determinants of biological and behavioural risk factors for cardiovascular diseases.

**Materials and methods:**

The present study was conducted among women in the age group of 15 to 49 years using nationally representative sample from fifth round National Family Health Survey in India. The data analysis in the current study included 7,24,115 women in the age group of 15 to 49 years. SPSS version 20 was used for the purpose of analysis. Weighted prevalence was computed for the studied behavioral and biological (dependent variable) risk factors using women specific weights as provided in the dataset. Binary logistic regression model was employed to calculate the adjusted odds ratio (OR) with the corresponding 95% confidence interval (CI) to study the sociodemographic determinants (independent variables) of these risk factors.

**Results:**

Highest prevalent risk factor for cardiovascular diseases was reported to be central obesity (78.2%), followed by overweight/obesity (23.9%), oral contraceptive use (13.4%), raised blood pressure (11.8%), raised blood sugar (8.6%), tobacco use (4.0%), and alcohol use (0.7%). Higher odds of all the studied risk factors were reported with increasing age. All of the studied risk factors, except for alcohol consumption [OR (95%CI): 0.9 (0.8–0.96)], had higher odds in rural areas compared to urban areas. Compared to other castes, the odds of tobacco [OR (95% CI): 2.01 (1.91–2.08)] and alcohol consumption [OR (95% CI): 5.76 (5.12–6.28)], and raised blood pressure [OR (95% CI): 1.07(1.04–1.11)] was significantly higher among the people belonging to schedule tribe.

**Conclusion and recommendation:**

The present study highlights the state-wise disparities in the burden and predictors of risk factors for cardio-vascular diseases among women of reproductive age. The study provides insights to these disparities, and focuses on the need of tailoring the disease prevention and control measures suiting to the local needs.

## Background

Rapid ongoing demographic transition happening in the country with economic growth and urbanization, has led to change in the epidemiological transition ratio across states, along with regional differences in the risk factors for these cardiovascular diseases [1]. In the year 2015, cardiovascular diseases (CVDs) have accounted for more than 2.1 million deaths in India [2]. Total deaths attributed to non-communicable diseases (NCDs), has increased in India from 61% on 2017 to 66% in the year 2022, as per the WHO NCD progress report [3, 4]. The country is witnessing a rampant increase in the NCD morbidity and mortality in the past decade, despite nearly a decade long launch and implementation of a dedicated program to curtail this epidemic of NCDs [5].

Long natural history of NCDs, including CVDs provide a large window of opportunity in identification, prediction and prevention of these diseases. Modifiable risk factors for CVDs include, behavioral (tobacco consumption, alcohol consumption, obesity, insufficient physical activity, and diet) and biological risk factors (raised blood sugar, and raised cholesterol) [6]. Apart from these established risk factors, few other factors that are specific to women contributing towards hypertension include, hormonal contraceptive use, pregnancy-related hypertension, age at menarche and menopause [7]. The distribution and diversity of these risk factors vary significantly among men and women. Also, women’s health is usually looked upon in terms of their reproductive health. Even though, cardio-vascular diseases are one of the leading causes of death and disability among women, globally as well as in India, the area of research is limited while considering cardiovascular health among women [8].

Literature has shown evidence of varied distribution of these risk factors across states, and rural and urban areas, with gradience in level of epidemiological transition happening across different states [1]. However, no study has addressed this issue at national and subnational levels. The objective of the study was to estimate the national and regional prevalence, and sociodemographic determinants of biological and behavioural risk factors for cardiovascular diseases among women of child bearing age across rural and urban India, using data from a nationally represented survey. Studying the distribution and determinants of these risk factors can help the policymakers in tailoring the program approaches to curb the impending epidemic of NCDs.

## Materials and methods

### Study settings

The present study was conducted using data from nationally representative sample of a cross-sectional survey [National Family Health Survey-5 (NFHS-5)] conducted in the year 2019–21. The study involves secondary data analysis from this survey. The survey was conducted by International Institute for Population Sciences (IIPS), under the headship of Ministry of Health and Family Welfare (MoHFW), India. The sample size was calculated taking into multiple considerations into account. Thus, producing indicators representative at district, state, and national level. The survey covered 707 districts, 28 states and 8 union territories. The data analysis in the current study included 7,24,115 women in the age group of 15 to 49 years.

### Sampling and data collection

Two-stage stratified cluster sampling was done for data collection, with stratification done at district level into rural and urban areas. The sampling frame for the survey was constructed using census 2011 for the selection of Primary Samling Units (PSU), which was taken as Census Enumeration Block (CEB) in the urban areas and villages in the rural areas. Considering the non-proportional allocation of the sample, sampling weights were computed, for adequate representation at all the levels. Details of the survey in terms of sampling design, selection of cluster in PSU, computation of sample weights, etc. is available in the country report. Out of 7,47,176 eligible women for the survey, 7,24,115 (97%) women completed the interview.

The data collection for the survey was conducted in two phases (phase 1 in June 2019 to January 2020 and phase 2 in January 2020 to April 2021). The survey collected data to provide indictors on population demography, health, and nutrition at district, state and national level. Data was collected for estimation of risk factors for non-communicable diseases including tobacco and alcohol consumption overweight/ obesity, waist and hip circumference, raised blood pressure, raised blood glucose, and oral contraceptive (OCP) use, which was used for the purpose of data analysis in the current study. Anthropometric measurements (height, weight, waist and hip circumference), blood pressure and random blood glucose were done for women age 15–49 years. Height and weight were measured using Seca 874 digital scale, and Seca 213 stadiometer, respectively. Waist and hip circumference were measured using Gulick tape. Blood pressure and blood sugar were measured using Omron Blood Pressure Monitor, and Accu-check Performa Glucometer, respectively. Three readings for blood pressure were recorded at 5 minutes interval and average of the three was considered for estimation of blood pressure. Wealth Index was computed at household level “based on the number and kinds of consumer goods they own, ranging from television to a bicycle or a car and housing characteristics such as source of drinking water, toilet facilities, and flooring materials.”

### Statistical analysis

The analysis for the present study was done for women between 15 to 49 years who completed the interview. Extreme values or outliers were excluded from the study before the analysis. Weighted prevalence was computed for all the risk factors using women specific weights as provided in the dataset. SPSS version 20 was used for the purpose of analysis. Binary logistic regression model was employed to calculate the adjusted odds ratio (OR) with the corresponding 95% confidence interval (CI). Collinearity between the independent variables was checked at the time of regression analysis.

Behavioral risk factors (including tobacco consumption, alcohol consumption, overweight/obesity, waist-hip ratio, and OCP consumption) and biological risk factors (including raised blood pressure, and raised blood sugar) were used as the dependent variable. Sociodemographic factors including age of the participant, marital status, educational status, wealth index, region, and caste were used as independent/ predictor variables.

### Operational definitions used

#### Tobacco use

Currently using any kind of smoked or smokeless tobacco.

#### Alcohol use

Currently having any kind of alcohol.

#### Overweight/obesity

Body mass index (BMI) of > 25 kg/m^2^.

#### Central obesity

Waist-hip ratio of more than 0.8.

#### Raised blood pressure

Hypertension was defined as “systolic blood pressure >= 140 mm of Hg or diastolic blood pressure >= 90 mm of Hg or history of current intake of antihypertensive medications.” Hypertension was computed using three variables i.e., average of the three systolic and diastolic blood pressure readings, and questions asked regarding the history of intake of any antihypertensive medications. In case of missing reading for any of the systolic or diastolic blood pressure, the average of the available two or one individual reading was taken to compute systolic and diastolic blood pressure.

#### Raised blood sugar

Raised blood sugar was defined as “random blood sugar of >140 mg/dl or history of current intake of medications for lowering blood sugar.”

#### Use of oral contraceptives

Use of oral contraceptive was defined as “ever use of the contraceptive including current and past use”.

## Results

### Sociodemographic profile of study participants

The study involved 7,24,115 women between the age of 15 to 49 years. The rural urban ratio of the study participants was nearly 2:1, with preponderance of the participants residing in rural India. Nearly 16% of the women were in the age group 15–19 years, 20–24 years, and 25–29 years, each. Almost 13% belonged to age group of 30–34 years and 35–39 years. Almost three-fourth of the women were ever married, out of whom 68.4% were from rural area, and rest 31.6% belonging to urban area. 22.4% of the study participants had no education, and almost 65% had education level of secondary school or higher. Majority of the study participants were Hindu by religion (81.4%) (Table 1).

**Table 1:** Sociodemographic profile of the study participants

Variables	Rural		Urban		Total	
	n	%	n	%	n	%
Age						
15–19	88,000	18.0	34,543	14.7	122,543	16.9
20–24	82,885	17.0	36,555	15.5	119,440	16.5
25–29	78,873	16.1	38,272	16.3	117,145	16.2
30–34	65,992	13.5	34,392	14.6	100,384	13.9
35–39	63,683	13.0	33,869	14.4	97,552	13.5
40–44	53,130	10.9	28,657	12.2	81,787	11.3
45–49	56,272	11.5	28,991	12.3	85,263	11.8
Marital Status						
Never married	111,033	22.7	61,041	25.9	172,074	23.8
Ever married	377,803	77.3	174,237	74.1	552,040	76.2
Education						
No education	132,805	27.2	29,646	12.6	162,451	22.4
Primary	63,842	13.1	21,081	9.0	84,923	11.7
Secondary	241,060	49.3	122,335	52.0	363,395	50.2
Higher	51,130	10.5	62,216	26.4	113,346	15.7
Caste						
Schedule Caste	112,893	24.2	45,590	20.5	158,453	23.0
Schedule tribe	57,554	12.3	9709	4.4	67,263	9.8
OBC	210,636	45.1	100,147	45.0	310,783	45.1
Others	89,598	18.4	67,072	30.1	153,030	22.2
Wealth Index						
Poorest	127,341	26.0	6632	2.8	133,973	18.5
Poorer	128,318	26.2	16,496	7.0	144,814	20.0
Middle	111,508	22.8	37,108	15.8	148,616	20.5
Richer	81,145	16.6	69,536	29.6	150,681	20.8
Richest	40,525	8.3	105,507	44.8	146,032	20.2
Religion						
Hindu	407,716	83.4	181,448	77.1	589,164	81.4
Muslims	57,057	11.7	40,538	17.2	97,595	13.5
Others	24,063	4.9	13,293	5.6	37,356	5.2
Regions						
North	65,296	13.4	36,904	15.7	102,200	14.1
Central	134,064	27.4	46,164	19.6	180,228	24.9
East	126,397	25.9	38,431	16.3	164,828	22.8
Northeast	21,488	4.4	5258	2.2	26,746	3.7
West	55,281	11.3	46,755	19.9	102,036	14.1
South	86,310	17.7	61,767	26.3	148,077	20.4
Total	488,836	67.5	235,279	32.5	724,115	100.0

*OBC* Other Backward Class

### Prevalence of risk factors for cardiovascular diseases

**Fig. 1:**
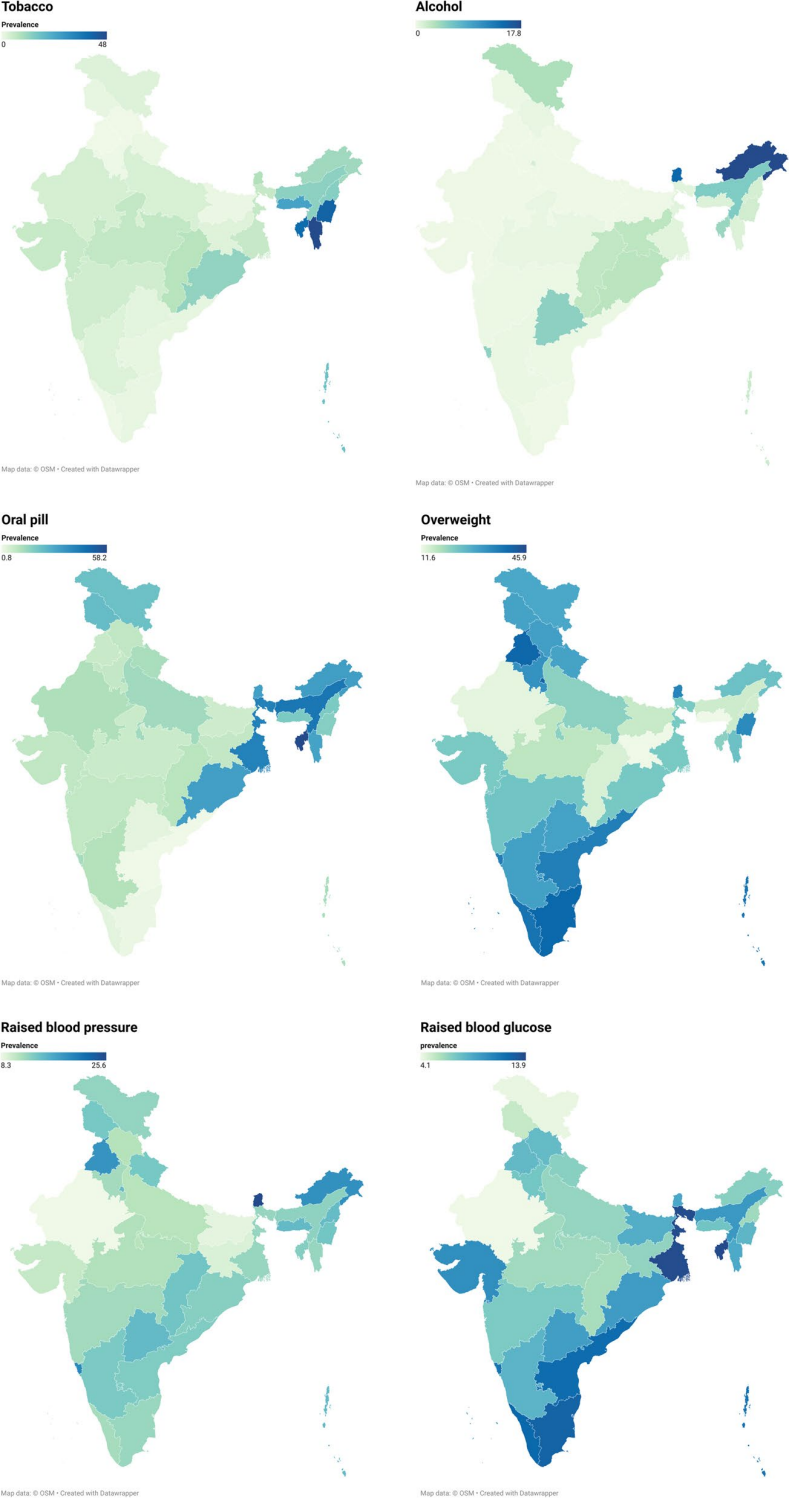
State-wise distribution of risk factors for cariovascular diseases

Highest prevalent risk factor for cardiovascular diseases was reported to be central obesity (78.2%; 77.2% in rural and 80.3% in urban), followed by overweight/obesity (23.9%; 19.5% in rural and 33.2% in urban), oral contraceptive use (13.4%; 13.6% in rural and 12.8% in urban), raised blood pressure (11.8%; 11.3% in rural and 12.9% in urban), raised blood sugar (8.6%; 8.1% in rural and 9.7% in urban), tobacco use (4.0%; 4.8% in rural and 2.5% in urban), and alcohol use (0.7%; 0.9% in rural and 0.4% in urban) (Table 2). State-wise prevalence of risk factors is shown in Fig. 1.

**Table 2:** Prevalence of various risk factors for cardiovascular diseases

	Rural [n (%)]	Urban [n (%)]	Total [n (%)]
Tobacco use	23,433 (4.8)	5883 (2.5)	29,316 (4.0)
Alcohol use	4409 (0.9)	991 (0.4)	5400 (0.7)
Raised Blood Pressure	50,525 (11.3)	26,834 (12.9)	77,359 (11.8)
Raised Blood Sugar	37,850 (8.1)	20,830 (9.7)	58,680 (8.6)
Overweight/ Obesity	92,507 (19.5)	72,582 (33.2)	165,089 (23.9)
Central Obesity	364,670 (77.2)	175,365 (80.3)	540,035 (78.2)
OCP use	52,022 (13.6)	22,641 (12.8)	74,663 (13.4)

*OCP* Oral Contraceptive pill

### Association of risk factors for cardiovascular diseases and sociodemographic profile of the study participants

All the conventional risk factors including alcohol use, tobacco use, oral contraceptive (OCP) use, raised blood pressure, raised blood glucose, overweight/obesity, and central obesity were found to be statistically significant with the socio-demographic independent variables on bivariate analysis (Table 3).

**Table 3:** Logistic regression analysis to study the association of risk factors for cardiovascular diseases and sociodemographic characteristics

Risk Factors [Adjusted OR (95% CI)]								
		Tobacco use	Alcohol use	Raised Blood pressure	Raised Blood glucose	Overweight and Obesity	Central obesity	OCP use
Age	15–19 years	1	1	1	1	1	1	1
	20–24 years	2.01(1.83–2.18)^*^	2.17(1.77–2.49)^*^	1.48(1.39–1.54)^*^	1.23(1.15–1.28)^*^	1.80(1.72–1.84)^*^	1.10(1.08–1.13)^*^	1.96(1.82–2.08)^*^
	25–29 years	2.72(2.49–2.98)^*^	3.08(2.53–3.6)^*^	2.09(1.94–2.16)^*^	1.62(1.55–1.73)^*^	2.97(2.87–3.09)^*^	1.25(1.22–1.29)^*^	2.85(2.63–3.01)^*^
	30–34 years	3.63(3.32–3.98)^*^	3.40(2.87–4.12)^*^	3.33(3.12–3.47)^*^	2.48(2.32–2.59)^*^	4.58(4.41–4.75)^*^	1.42(1.40–1.48)^*^	3.23(2.99–3.41)^*^
	35–39 years	4.52(4.14–4.95)^*^	4.35(3.59–5.12)^*^	5.02(4.75–5.27)^*^	3.30(3.09–3.45)^*^	5.73(5.46–5.89)^*^	1.60(1.55–1.64)^*^	3.18(2.97–3.39)^*^
	40–44 years	5.50(5.02–6.01)^*^	4.99(4.13–5.91)^*^	7.74(7.29–8.18)^*^	4.87(4.59–5.12)^*^	6.52(6.25–6.75)^*^	1.89(1.83–1.94)^*^	2.83(2.61–2.99)^*^
	45–49 years	6.23(5.65–6.76)^*^	5.16(4.26–6.09)^*^	10.86(10.23–11.36)^*^	6.36(5.97–6.66)^*^	6.60(6.37–6.89)^*^	2.19(2.11–2.25)^*^	2.48(2.31–2.65)^*^
Marital Status	Ever married	1	1	1	1	1	1	1
	Never married	0.76(0.70–0.80)^*^	1.42(1.21–1.52)^*^	0.98(0.93–1.01)	0.89(0.85–0.92)^*^	0.56(0.54–0.57)^*^	0.74(0.72–0.76)^*^	0.51(0.45–0.57)^*^
Education	No education	1	1	1	1	1	1	1
	Primary	1.01(0.95–1.02)	0.47(0.42–0.5)^*^	1.09(1.05–1.11)^*^	1.18(1.14–1.21)^*^	1.26(1.23–1.29)^*^	1.03(1.01–1.06)^*^	1.50(1.46–1.55)^*^
	Secondary	0.45(0.44–0.47)^*^	0.31(0.28–0.34)^*^	0.98(0.96–1.01)	1.15(1.12–1.18)^*^	1.38(1.35–1.4)^*^	1.10(1.08–1.12)^*^	1.63(1.61–1.68)^*^
	Higher	0.15(0.14–0.17)^*^	0.47(0.40–0.52)^*^	0.80(0.77–0.82)^*^	0.96(0.92–0.99)^*^	1.31(1.30–1.36)^*^	1.19(1.16–1.22)^*^	1.22(1.19–1.28)^*^
Residence	Rural	1	1	1	1	1	1	1
	Urban	1.30(1.23–1.33)^*^	0.91(0.80–0.96)^*^	1.06(1.04–1.09)^*^	1.04(1.02–1.06)^*^	1.20(1.19–1.23)^*^	1.20(1.18–1.22)^*^	1.11(1.09–1.14)^*^
Wealth Index	Poorest	1	1	1	1	1	1	1
	Poorer	0.71(0.70–0.75)^*^	0.50(0.45–0.53)^*^	1.06(1.03–1.08)^*^	1.06(1.02–1.08)^*^	1.60(1.56–1.64)^*^	1.03(1.01–1.05)^*^	1.16(1.13–1.19)^*^
	Middle	0.57(0.55–0.59)^*^	0.43(0.38–0.47)^*^	1.15(1.12–1.18)^*^	1.13(1.09–1.17)^*^	2.25(2.19–2.31)^*^	1.12(1.09–1.14)^*^	1.16(1.12–1.19)^*^
	Richer	0.35(0.34–0.37)^*^	0.36(0.32–0.41)^*^	1.22(1.18–1.25)^*^	1.30(1.26–1.35)^*^	2.99(2.89–3.05)^*^	1.20(1.19–1.24)^*^	1.11(1.06–1.17)^*^
	Richest	0.22(0.21–0.24)^*^	0.66(0.55–0.72)^*^	1.28(1.23–1.32)^*^	1.39(1.32–1.43)^*^	4.08(3.97–4.20)^*^	1.31(1.27–1.34)^*^	1.09(1.03–1.11)^*^
Region	South	1	1	1	1	1	1	1
	North	1.44(1.32–1.5)^*^	0.23(0.21–0.27)^*^	0.96(0.94–0.99)^*^	0.53(0.51–0.55)^*^	0.60(0.58–0.61)^*^	1.73(1.70–1.78)^*^	2.90(2.74–2.96)^*^
	Central	2.31(2.18–2.4)^*^	0.30(0.27–0.33)^*^	1.04(1.03–1.08)^*^	0.70(0.68–0.72)^*^	0.64(0.63–0.65)^*^	1.18(1.16–1.21)^*^	3.56(3.38–3.63)^*^
	East	1.90(1.79–1.98)^*^	0.63(0.58–0.68)^*^	0.92(0.91–0.96)^*^	1.14(1.11–1.17)^*^	0.68(0.67–0.69)^*^	2.34(2.29–2.38)^*^	7.49(7.23–7.71)^*^
	Northeast	11.01(10.38–11.69)^*^	4.27(3.82–4.58)^*^	1.19(1.11–1.22)^*^	1.10(0.99–1.14)	0.65(0.62–0.67)^*^	2.24(2.15–2.35)^*^	15.12(14.4–15.8)^*^
	West	3.10(2.92–3.25)^*^	0.13(0.12–0.17)^*^	0.88(0.85–0.9)^*^	0.74(0.72–0.77)^*^	0.51(0.51–0.52)^*^	0.65(0.64–0.66)^*^	2.08(1.98–2.14)^*^
Caste	Others	1	1	1	1	1	1	1
	Schedule Caste	1.14(1.10–1.19)^*^	1.13(1.04–1.26)^*^	0.92(0.91–0.95)^*^	0.91(0.90–0.95)^*^	0.80(0.80–0.83)^*^	0.96(0.95–0.98)^*^	0.90(0.88–0.92)^*^
	Schedule tribe	2.01(1.91–2.08)^*^	5.76 (5.12–6.28)^*^	1.07(1.04–1.11)^*^	0.80(0.77–0.83)^*^	0.57(0.56–0.59)^*^	0.93(0.92–0.96)^*^	0.79(0.76–0.81)^*^
	OBC	0.83(0.81–0.87)^*^	0.99 (0.86–1.07)	0.90(0.89–0.93)^*^	0.91(0.88–0.93)^*^	0.80(0.78–0.81)^*^	0.90(0.88–0.91)^*^	0.75(0.73–0.77)^*^

*OR* Odds Ratio, *CI* Confidence Interval, *OCP* Oral Contraceptive Pills, *OBC* Other Backwards Classes, *p-value: < 0.05

Variable adjusted in the regression analysis included age, marital status, education, residence, Wealth Index, Region, and Caste

Higher odds of all the studied risk factors were reported with increasing age. The odds of alcohol consumption [OR (95%CI): 1.42(1.21–1.52)] was significantly higher among never married women compared to ever married women. However, odds of OCP use [OR (95%CI): 0.51(0.45–0.57)], tobacco [OR (95%CI): 0.76(0.70–0.80)], raised blood sugar [OR (95%CI): 0.89(0.85–0.92)], overweight/obesity [OR (95%CI): 0.56(0.54–0.57)], and central obesity [OR (95%CI): 0.74(0.72–0.76)] was significantly lower among never married women (Table 3).

Decreasing odds of alcohol and tobacco use, and raised blood pressure was observed with increasing educational status. Significantly higher odds of OCP use [OR (95%CI): 1.63(1.61–1.68)], raised blood sugar [OR (95%CI): 1.15(1.12–1.18)], overweight/obesity [OR (95%CI): 1.38(1.35–1.40)], and central obesity [OR (95%CI): 1.10(1.08–1.12)] was observed among those who had secondary level of education compared to those who were uneducated (Table 3).

All of the studied risk factors, except for alcohol consumption [OR (95%CI): 0.91(0.80–0.96)], had higher odds in rural areas compared to urban areas. Increasing odds of risk factor including raised blood pressure, raised blood glucose, overweight/obesity, central obesity, and OCP use, was observed with rising wealth index quartile (*p*-value: < 0.01) (Table 3). The odds of tobacco consumption were significantly higher within other regions compared to southern region, with highest odds reported in the north-eastern states [OR (95%CI): 11.01(10.38–11.69)]. Also, higher odds of alcohol consumption, were reported in the north-eastern states compared to southern states [OR (95%CI): 4.27(3.82–4.58)]. Central obesity [OR (95%CI): 2.24(2.15–2.35)]and OCP consumption [OR (95%CI): 15.12(14.4–15.8)] was significantly higher in these states compared to southern states (Table 3).

Compared to other castes, the odds of tobacco [OR (95% CI): 2.01(1.91–2.08)] and alcohol consumption [OR (95% CI): 5.76(5.12–6.28)], and raised blood pressure [OR (95% CI): 1.07(1.04–1.11)] was significantly higher among the people belonging to schedule tribe. However, the odds of raised blood glucose [OR (95% CI): 0.80(0.77–0.83)], overweight/ obesity [OR (95% CI): 0.57(0.56–0.59)], central obesity [OR (95% CI): 0.93(0.92–0.96)], and OCP use [OR (95% CI): 0.79(0.76–0.81)] was significantly lower among the people belonging to schedule tribe. (Table 3).

## Discussion

The study involves secondary data analysis of modifiable risk factors for CVDs among 15 to 49 years age women, from a nationally representative sample of India. As majority of the non-communicable diseases is preceded by common risk factors, assessment of these risk factors will help in prevention and control of NCDs in future. Our study will help in providing in depth understanding of the regional diversity and predictors of risk factors for CVDs among women in India. Health is a state subject in India. Thus, the findings in our study can help the program managers to remould the program components as per their regional needs.

Overall tobacco consumption (including both smoking and smokeless tobacco) in the country among women was 4%, with higher proportion reporting tobacco use in rural (4.8%) India compared to urban (2.5%) India. Similar findings were also reported by studies done in different regions of the country [9–11]. Although overall tobacco consumption was higher in the rural areas, studies have reported higher consumption of smoking in urban areas and smokeless tobacco in the rural areas [10, 11], which could be attributed to easy availability and lower cost of smokeless tobacco. Also, even though, the overall prevalence of tobacco consumption was higher among people living in the rural areas, the risk of tobacco consumption was found to be higher among those living in urban areas, after adjusting for other confounders. The range of tobacco consumption across various states was found to vary from as low as no consumption in Chandigarh to as high as 48% in Mizoram. North-eastern states including Assam, Nagaland, Meghalaya, Tripura, Manipur and Mizoram reported very high consumption of tobacco compared to the national average. The same can be attributed to its high social acceptability and its cultural integration. The findings have been same even in the previous round of the national survey [12]. Tobacco consumption was significantly higher among older age, lower educational status, and participants belonging to poorer wealth index bracket. Higher accessibility and availability to tobacco and tobacco products with increasing age could be attributed to its higher consumption among them.

Alcohol consumption has been reported to raise the likelihood of multi-morbidity among women of reproductive age by 18% [13]. Alcohol consumption in the current study ranged from zero (Lakshadweep) to 17.8% (Arunachal Pradesh), with a national average of 0.7%. This wide variance in the pattern of alcohol consumption could be attributed to regional socio-cultural differences, extent of accessibility and availability. This variability across different states can also be due to state-wise differences in manufacturing, and sales regulations. Very high prevalence of alcohol consumption among women in Mizoram and Manipur could be attributed to lifting on ban on liquor nearly after two decades, with increasing accessibility and social acceptability of its use [14, 15]. Also, reason behind lifting of this ban on liquor prohibition was said to be due to incidences of adverse health events due to illicit liquor consumption. This thus, acts as a double-edged sword, and unveil the need for amendment in regulation to balance sale and utilization of alcohol in these states. Older age, never married women, and lower education level, had higher odds of alcohol consumption in our study. Increasing age provide a window of ease in accessibility and also, societal pressure of social drinking with rapid urbanization, could be the reason for its higher prevalence among older age, never married and employed women. Thus, despite of lower rates of alcohol use in majority of the Indian states, addressing the pattern of alcohol consumption still sustains as a public health priority.

Overweight/obesity was second most common CVD risk factor (23.9%), with urban areas reporting higher burden (33.2%) compared to rural areas (19.5%). Although, urban areas reported a higher burden, with the epidemiological transition happening across the country, and expansion of urban and peri-urban areas, the rise in the overweight/obesity among women is expected to be higher among the rural compared to the urban women by 2040 [16]. Inadequate dietary practices, lack of physical activity, and easy availability to unhealthy diet could be the reason behind such findings. Overweight/obesity ranged from 11.6% to 45.9% across different states. The odds of overweight/obesity were reported to be increasing with increasing age and educational status in the current study. Similar findings were also reported in a study done in South India, but, the prevalence was reported to be higher compared to our findings [16, 17]. Ever married women reported to have higher odds of overweight/obesity compared to never married women. However, conflicting findings were reported by study a cross-sectional survey done in Punjab [18]. The difference in the findings could be attributed to difference in the categorization of marital status. Also, the later study considered only obesity while computation of possible predictors.

High waist-hip ratio has been reported to be a major predictor of coronary artery disease [19]. Central obesity was reported to be the most common CVD risk factor in our study, with an overall prevalence of 78.2%, with higher prevalence in urban areas compared to the rural areas. Very high prevalence of central obesity was reported in all the states ranging from 64.4% in Madhya Pradesh to 97% in Jammu & Kashmir. Almost similar findings were reported by study conducted in north-western Iran [20]. Such high burden of central obesity could be attributed to easy availability of cheap calorie dense junk foods, and lack of physical activity. Older age was reported to be a significant predictor of central obesity, which has also been reported in other studies [20, 21]. This could also be attributed to few gender specific factors like weight gain following menopause, age at menarche, social and cultural restriction among women limiting their physical activity, etc. [22].

Globally, hypertension accounts for 57 million disability adjusted life years (DALYs), which constitutes 3.7% of the DALYs [1]. Prevalence is higher among low- and middle-income countries in comparison to high income countries [23]. The overall prevalence of raised blood pressure in the present study was 11.8%, with almost similar distribution in the rural and urban areas. However, a higher prevalence has been reported in other South-east Asian countries [24, 25]. The odds of raised blood pressure was reported to be higher with increasing age. Age is an established predictor of raised blood pressure, as also reported by other studies [26, 27].

Diabetes is an established precursor of multiple chronic diseases such as heart attack, stroke, renal failure, blindness, nerve damage and premature death and disability [28]. High fasting plasma glucose was responsible for 6% of DALYs in India [1]. Raised blood sugar was reported to be 8.6% in the country, with almost equal distribution in the rural and urban areas, ranging from 4.1% in Rajasthan to 13.9% in Tripura. Higher prevalence was reported in study conducted in Kerala (raised fasting sugar: 19.2%), and another nation-wide NCD risk factor survey [29, 30]. Reason behind such varied findings could be due to different cut offs taken by different studies and differences in the population studied. The current study reported significantly higher prevalence of raised blood glucose among older age, which was in agreement with other studies conducted in the country [18, 27].

Oral contraceptives have been reported to increase the risk of ischemic heart disease, stroke and hypertension [31, 32]. Nation-wide prevalence of oral contraceptive use was found to be 13.4%. Wide disparity in the OCP consumption was reported across various states with a prevalence of as low as 0.8% in Andhra Pradesh, and as high as 58.2% in Tripura. Highest odds of OCP consumption were reported in the north-eastern states compared to the southern states. The possible reason for this variation could be state-wise difference in the availability and accessibility to contraceptives and introduction of other newer methods in the programme. Highest odds of OCP use were reported among women ageing 25 to 39 years of age, which constitutes majority of the eligible couples. Adoption of newer other methods of contraception and OCP consumption following risk assessment should be incorporated to address the CVD disease burden attributed to OCP use.

## Conclusion and recommendation

The present study highlights the state-wise disparities in the burden of risk factors for CVDs among women of reproductive age women, using a nationwide survey. The study provides insights into the disparities as per the various studied sociodemographic characteristics including, age of the participant, marital status, educational status, socio-economic status, region, and caste and focuses on the need of tailoring the disease prevention and control measures suiting to the local needs. Also, the study highlights the need for evaluation of the temporal association of these factors using longitudinal study designs.

### Limitations

Owing to the cross-sectional nature of the study design, temporality and causality of these predictors for CVD risk factors could not be assessed. As the study was done using secondary data set, inclusion of other possible independent predictors, was a limitation for the present study.

## Data Availability

The original dataset of NFHS-5 cannot be accessed from an open source. However, the dataset is available from DHS program on request, using the following link: https://www.dhsprogram.com/data/available-datasets.cfm.

## Abbreviations

BMI: Body Mass Index
CEB: Census Enumeration Block
CI: Confidence Interval
CVDs: Cardiovascular Diseases
IIPS: International Institute for Population Sciences
MoHFW: Ministry of Health and Family Welfare
NCDs: Non-communicable Diseases
NFHS: National Family Health Survey
OCP: Oral Contraceptive Pill
OR: Odds Ratio
PSU: Primary Sampling Unit
